# The Impact of Health Behaviours on Incident Cardiovascular Disease in Europeans and South Asians – A Prospective Analysis in the UK SABRE Study

**DOI:** 10.1371/journal.pone.0117364

**Published:** 2015-03-02

**Authors:** Anne Eriksen, Therese Tillin, Laura O’Connor, Soren Brage, Alun Hughes, Jamil Mayet, Paul McKeigue, Peter Whincup, Nish Chaturvedi, Nita G. Forouhi

**Affiliations:** 1 Medical Research Council Epidemiology Unit, University of Cambridge School of Clinical Medicine, Institute of Metabolic Science, Cambridge Biomedical Campus, Cambridge, United Kingdom; 2 University College London Institute of Cardiovascular Science, University College London, London, United Kingdom; 3 International Centre for Circulatory Health, St Mary’s Hospital, Paddington, Imperial College London, London, United Kingdom; 4 Centre for Population Health Sciences, University of Edinburgh Medical School, Edinburgh, United Kingdom; 5 Population Health Research Centre, St George's, University of London, London, United Kingdom; Weill Cornell Medical College in Qatar, QATAR

## Abstract

**Background:**

There is consistent evidence on the impact of health behaviours on risk of cardiovascular disease (CVD) in European populations. As South Asians in the UK have an excess risk of CVD and coronary heart disease (CHD) compared to Europeans, we investigated whether a similar association between combined health behaviours and risk of CVD and CHD among this high-risk group exists, and estimated the population impact.

**Methods and Findings:**

In a prospective cohort of 1090 Europeans and 1006 South Asians (40–69 y) without prevalent CVD at baseline (1988–1990), followed up for 21 years to 2011, there were 601 incident CVD events [Europeans n = 255; South Asians n = 346] of which 520 were CHD events [n = 207 and 313 respectively]. Participants scored between 0 to 4 points for a composite score including four baseline healthy behaviours (non-smoker, moderate alcohol intake, physically active, frequent fruit/vegetable intake). Adjusted hazard ratios (95% confidence intervals) for incident CHD in Europeans who had three, two, one, and zero compared to four health behaviours were 1.33 (0.78–2.29), 1.96 (1.15–3.33), 1.36 (0.74–2.48) and 2.45 (1.18–5.10), respectively, *p*-trend = 0.025. In South Asians, corresponding HRs were 2.88 (1.33–6.24), 2.28 (1.06–4.91), 3.36 (1.53–7.39) and 3.48 (1.38–8.81), *p*-trend = 0.022. The results were similar for incident CVD; Europeans HR 2.12 (1.14–3.94), *p*–trend = 0.014; South Asians HR 2.73 (1.20–6.21), *p*-trend = 0.018. The population attributable fraction in Europeans was 43% for CHD and 28% for CVD. In South Asians it was 63% and 51% respectively.

**Conclusions:**

Lack of adherence to four combined health behaviours was associated with 2 to 3-fold increased risk of incident CVD in Europeans and South Asians. A substantial population impact in the South Asian group indicates important potential for disease prevention in this high-risk group by adherence to healthy behaviours.

## Introduction

Cardiovascular diseases (CVD), particularly coronary heart disease (CHD) and stroke, are the leading cause of mortality globally, estimated to account for 17 million deaths (23% of total) annually [1, 2]. Epidemiological evidence supports a potentially protective role of individual healthy lifestyle behaviours on CVD risk, including smoking status [3, 4], alcohol intake [5–7], physical activity [8, 9] and dietary intake of fruit and vegetables [10–13]. There is accumulating evidence that due to the likely clustering of lifestyle behaviours, both research and public health efforts should shift to assessing the association of combined health behaviours with health outcomes.

An inverse association between combined health behaviours and CVD outcomes has been reported previously among mainly European origin populations [14–18]. These studies investigated the combined effect of smoking, alcohol intake, physical activity, diet and BMI on incident CHD in men [15], and in women [14], incident stroke in women [16], and (without BMI) incident stroke in men and women [18]. One Japanese study investigated the combined effect of these and a further four health behaviours on CVD mortality in men and women [17]. Substantial population impact of health behaviours on CVD outcomes has also been reported [14, 15, 19]. Though different populations share similar risk factors for CVD [20–22], the association and potential impact of combined health behaviours on CVD risk is unclear among immigrant South Asians, a group with up to 2-fold higher risk of CHD compared to European populations [23–26].

Our study had two objectives. First, to prospectively examine the association between a composite health behaviour score of four healthy behaviours (non-smoker, moderate alcohol intake, physically active, and frequent fruit and vegetable intake) and incident CVD and CHD among Europeans and South Asians living in the UK. Second, to estimate the population impact of combined health behaviours on the burden of these diseases among the two ethnic groups, using the population attributable fraction (PAF). A secondary objective was to examine the association of individual health behaviours with CVD and CHD incidence in both ethnic groups.

## Methods

The tri-ethnic cohort study, the Southall and Brent Revisited (SABRE), was established in 1988–1991, with a population sample of men and women aged 40–69 years at baseline, followed for morbidity and mortality to 2008–2011 [27]. The current analyses are based on the Southall arm of the SABRE study, which recruited 1,761 Europeans and 1,710 South Asians. Recruitment was through age-sex stratified lists of general practices and industrial workforces in West London with response rates of 66% and 62% among Europeans and South Asians respectively [28]. The baseline studies were initially designed to study ethnic differences in metabolic risk factors in association with cardiovascular disease (CVD) in men, hence there is a male preponderance in the sample. South Asians were defined as people with ancestral origins in India, Pakistan, or Bangladesh and Europeans with UK or other European ancestral origin. Ethnic origin was ascertained on the basis of name, country of birth, appearance, and further enquiry in case of doubt. All participants gave written informed consent. Approval for the study at baseline was obtained from ethics committees at Ealing, Hounslow and Spelthorne and University College London, and at follow-up from St Mary’s Hospital Research Ethics Committee (ref. 07/H0712/109).

**Fig 1:**
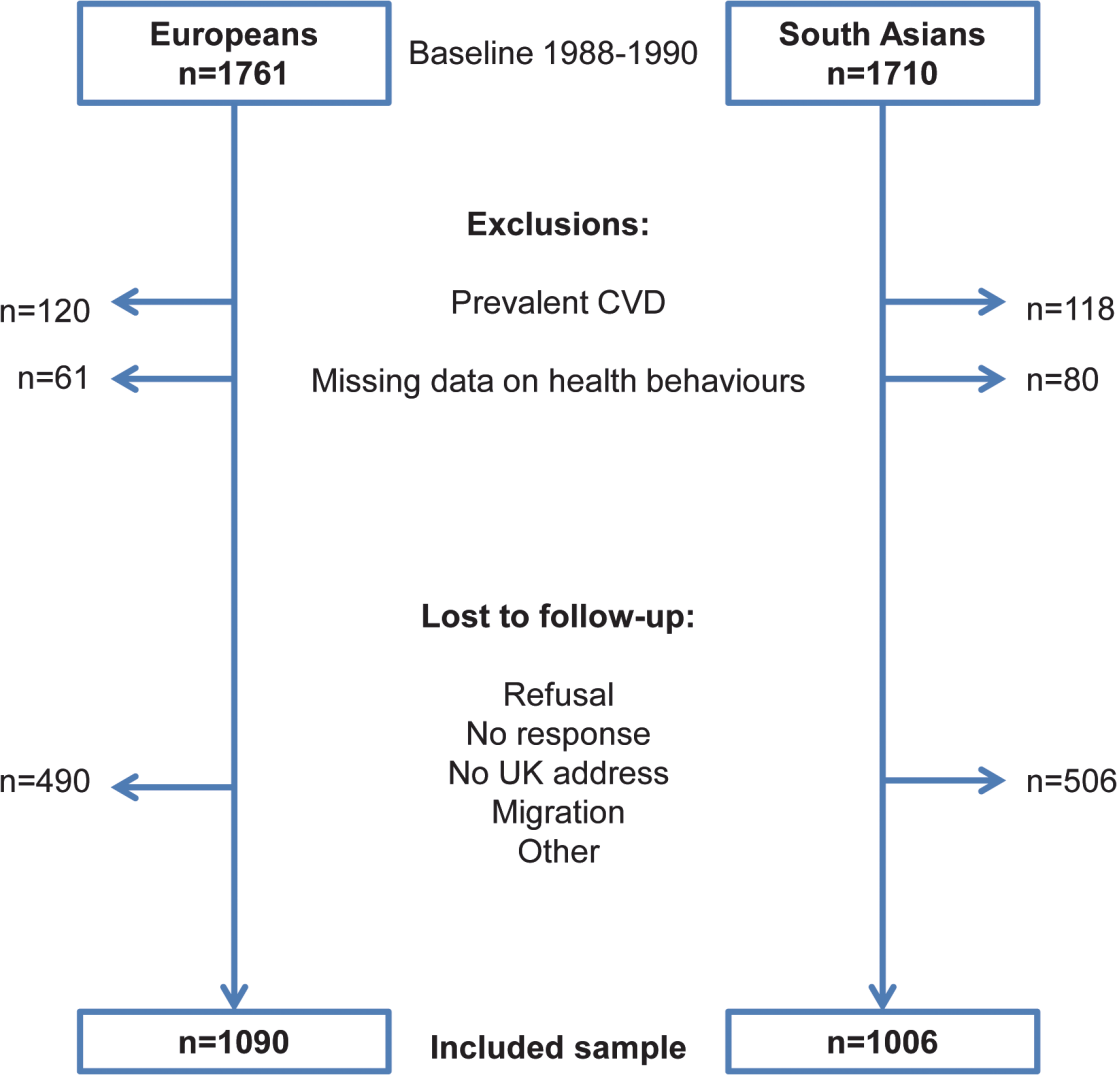
Flow diagram of exclusions and follow-up of participants from the SABRE study, UK.

For the present investigation, we excluded participants with prevalent CHD or stroke (n = 120 Europeans; n = 118 South Asians) and participants with missing health behaviour data (n = 61; n = 80). We also excluded those without direct follow-up data from either death certificate, medical record review, clinic visit or questionnaire (n = 490 Europeans; n = 506 South Asians), **Fig. 1**. The sample for current analyses included 1,090 Europeans (86% men) and 1,006 South Asians (83% men).

## Data Collection

### Baseline measurements

Participants were examined at the research clinic using standardised protocols. Waist and hip circumferences and height and weight were measured and BMI calculated as weight in kilograms divided by height in metres squared. The mean of two seated resting blood pressure readings (mmHg) was used. Fasting blood samples were drawn and non-diabetic participants were given a 75 g oral glucose tolerance test [27]. Glucose and lipids were analysed using standardised assays. Prevalent diabetes was defined according to World Health Organisation criteria [28], self-report of physician diagnosed diabetes, or receipt of anti-diabetes medications.

### Baseline questionnaire-based data

A self-administered questionnaire included information on medical history, smoking status, alcohol intake, diet, physical activity, occupation, and socio-demographic background. Social class was classified according to the Registrar General’s occupation-based classification [29] into five main categories, and categorised into non-manual and manual occupations. Smoking status was derived from yes/no responses to the questions “Do you smoke cigarettes?” and “Have you ever smoked cigarettes regularly?” and categorised into never, former and current smokers. Alcohol intake was derived from a series of questions; “In the past 12 months have you taken an alcoholic drink:” followed by answer categories from twice a day to not at all; “How often have you had a drink of beer or cider during the last 12 months?” and “When you had a drink of beer or cider in the last 12 months, how many pints have you usually drunk on any occasion?” was used to derive the number of units consumed on average per week. This was done similarly for wine, fortified wine and spirits. A unit of alcohol was defined as half a pint of beer or cider, a glass of wine or fortified wine, or a single unit of spirits. Physical activity was derived from participant responses to questions about usual physical activity based on the Allied Dunbar National Fitness Survey questionnaire [30]. This included sections on occupational activity, commuting (walking and cycling), sports and other strenuous activity. Physical activity energy expenditure was calculated as a product of frequency, duration and intensity (in metabolic equivalent task units, METs) for each specific activity [31]. Frequency of fruit and vegetable consumption was assessed using a simple dietary questionnaire with response categories of ‘not in the last week’, ‘once in the last week’, ‘2–3 times in the last week’, and ‘on most days in the last week’ for the intake of “fresh green vegetables” and “fresh fruit”.

### Health behaviour score

Four health behaviours (smoking, alcohol consumption, physical activity level and fruit and vegetable consumption) were categorised dichotomously into healthy and unhealthy as follows.

A healthy smoking behaviour was defined as not currently smoking. A moderate alcohol intake was defined as consuming between one unit per week and up to 14 units/week for women or 21 units/week for men based on past evidence on alcohol consumption and CVD outcomes among Europeans [5–7]. As alcohol consumption patterns differ between Europeans and South Asians, with abstention being more common among the latter [32], we performed an exploratory analysis to test the appropriateness of the currently used cut-off among both ethnic groups. When we compared abstention with our definition of moderate alcohol intake we found increased hazard of CVD in both Europeans and South Asians (HR: 1.53 (1.11–2.11) and 1.31 (1.00–1.71), respectively), independent of age, sex, BMI, hypertension, lipids and social class, supporting the use of the cut-off in both ethnic groups. Physical activity was summarised as the total weekly energy (MJ) expended in sporting activities, cycling, walking, and in other strenuous activity during leisure time. Physically active was defined as those undertaking moderate exercise (e.g. walking, cycling, gardening) for 5 h or more per week or vigorous exercise (e.g. jogging, swimming) for ≥2.5 h per week, which equals a daily activity energy expenditure of at least 3 kcal/kg/day. This cut-off was based on ethnic specific data [33] where physical activity was categorised as moderately to highly active when energy expenditure was ≥3 kcal/kg/day, a level adequate to obtain health benefits [34]. A frequent (healthy) fruit and vegetable intake was defined as consumption of fruit or vegetables on most days, with intakes of ≥5.5 times/week of either fruit, or vegetables, or both.

A combined health behaviour score was constructed ranging from a minimum score of zero to a maximum score of four. Participants scored one point if they complied with a healthy behaviour and zero if they did not.

### Case ascertainment

Between 2008–2011, surviving participants were followed-up through a combination of completing health and lifestyle questionnaires, primary care medical record review and/or attendance at a study clinic visit [30]. All participants were flagged for mortality with the Office of National Statistics (ONS). For CHD, we included the first event after baseline identified from cause of death including any of the following: angina, myocardial infarction or its sequelae or atherosclerotic heart disease using International Classification of Disease (ICD) 9 codes 410–415 or ICD10 codes I200-I259 [35]. Primary care data were independently reviewed by two senior physicians blinded to participant ethnicity and identity. Adjudication by a third physician was conducted if required. For stroke, the first event after baseline was identified from cause of death including any of the following ICD9 codes 430–439 or ICD10 codes I600-I698. Primary care data were reviewed in a similar manner to CHD, with definite or probable diagnosis of stroke made according to pre-determined criteria based on symptoms, duration of symptoms and MR/CT imaging. We also included participant report of physician diagnosed stroke and duration of symptoms in excess of 24 hours [27]. We combined any first post-baseline fatal or non-fatal CHD or stroke event as CVD outcome.

### Statistical analyses

Analyses were performed using Stata/SE 12.1 (Stata Corp, College Station, TX, USA). Baseline characteristics were summarised by the number of health behaviours for each ethnic group separately using means ± SD, medians (IQR) and frequencies and percentages as relevant. Differences were tested using ANOVA, Kruskal-Wallis, or χ^2^ tests, as appropriate.

There was no interaction between sex and health behaviours on CVD events, but a significant interaction (*p*<0.001) was present between ethnicity and health behaviour score, justifying combined analyses for men and women but separate analyses by ethnicity. After checking that the proportional hazards assumption was not violated, we used Cox proportional hazards regression with calendar time (years) as the underlying timescale to examine the association between combined and individual health behaviours and incident CVD. We also examined incident CHD as an outcome. Each individual contributed person-time from the year of entry into the study until the year of first fatal or non-fatal event of either CHD or stroke, year of death from any other cause, or end of follow-up in November 2011, whichever came first.

For the combined health behaviours two models were constructed; Model 1 adjusted for age and sex; Model 2 was additionally adjusted for potential confounders and major CVD risk factors: systolic and diastolic blood pressure, hypertension treatment, total cholesterol, HDL-cholesterol, BMI, social class, employment status, and occupational physical activity. For the individual health behaviours a third model (Model 3) was constructed which was the same as Model 2 above but additionally mutually adjusted for the three remaining health behaviours.

A number of sensitivity analyses were conducted by separately including in model 2 each of: education, prevalent diabetes, triglycerides, waist circumference, and waist to hip ratio. We also excluded participants with events in the first two years and in the first five years of follow-up to minimise potential bias from reverse causality.

We applied several approaches to address potential bias due to missing data. To test whether the participants with and without follow-up data differed, we compared baseline characteristics between the total sample, those with follow-up data, and those without follow-up data. We also examined the association between health behaviours and fatal CVD in the total sample (n = 3,088), including those with baseline data (without prevalent CVD) on health behaviours. This approach allowed us to include also those lost to follow-up. Finally, using logistic regression we examined the association between missing follow-up data (yes/no) and number of baseline health behaviours in the total sample.

We calculated the PAF [36] to estimate the proportion of CVD and CHD cases within this population that could have been avoided had all participants adhered to the healthy behaviours, assuming a causal relation between exposure and outcome. To calculate the PAF we compared participants with a health behaviour score of 4 with the rest of the population in each ethnic group using adjusted Cox regression (Model 2) and the Rockhill et al. method [36]:

$$PAF=1-\sum_{i=0}^{k}\frac{pdi}{RRi}$$

where pd_i_ is the proportion of total cases in the population in the *i*th exposure category and RR_i_ is the adjusted relative risk for the *i*th exposure category relative to the unexposed (those with all 4 health behaviours). This equation allows multi-category exposures to be considered, rather than comparing only the extremes.

## Results

During a median of 21 (IQR 17–22) years of follow-up with 37,746 person-years, there were 601 CVD events [n = 255 in Europeans; n = 346 in South Asians], including 261 fatal CVD events [n = 115; n = 146]. Among 601 CVD events, 520 were from CHD [n = 207 in Europeans; n = 313 in South Asians], including 218 fatal CHD events [n = 92; n = 126]. CVD incidence rates were 12.7 per 1000 person years in Europeans and 19.6/1000 in South Asians, whereas CHD incidence rates were 10.1/1000 and 17.4/1000 person years, respectively.

**Fig 2:**
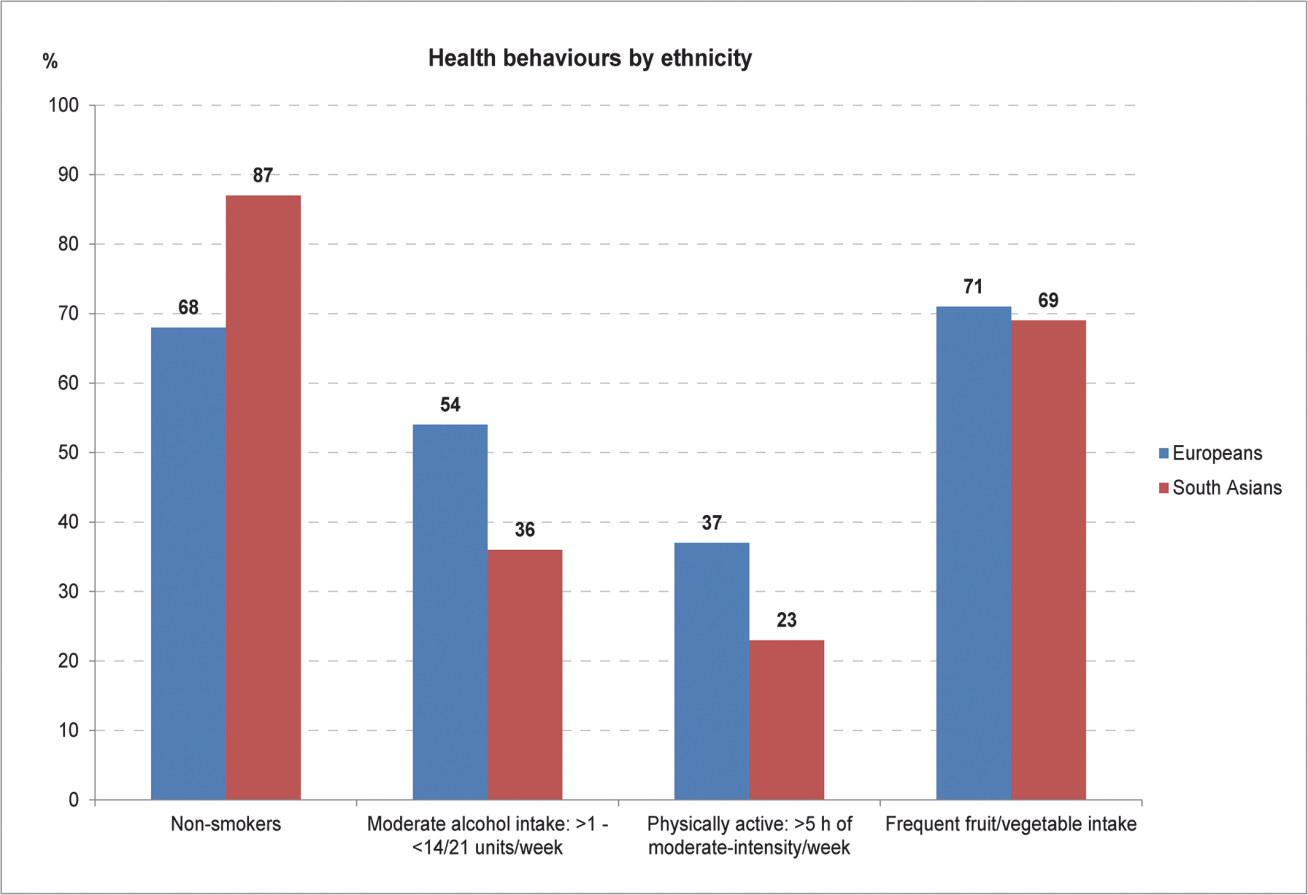
Distribution of four individual health behaviours by ethnicity; the SABRE study, UK.

Being a non-smoker and having frequent fruit and vegetable intake were the most common healthy behaviours in both Europeans and South Asians (**Fig. 2**). South Asians were less physically active and less likely to have a moderate alcohol intake than Europeans. Physical activity contributed least to the health behaviour score in both groups; 37% of Europeans and 23% of South Asians were categorised as physically active. Overall, adherence to all four health behaviours was observed in 12% of Europeans and 5% of South Asians, whereas 6% of Europeans and 3% of South Asians had zero healthy behaviours (**Table 1**). With greater adherence to health behaviours, there was higher physical activity and fruit and vegetable intake but lower alcohol intake and smoking among Europeans, while in South Asians, only smoking frequency was lower.

**Table 1:** Baseline characteristics by number of health behaviours of 1,090 Europeans and 1,006 South Asians aged 40–69 (without prevalent cardiovascular disease) at baseline (1988–1990); the SABRE study, UK.

	European						South Asians					
	Number of health behaviours						Number of health behaviours					
Variable	0	1	2	3	4	P value*	0	1	2	3	4	P value*
Number (n)	60	176	347	376	131		32	170	481	267	53	
Age (years)	50.9±6.9	53.2±7.3	53.2±7.3	52.9±7.2	53.1±7.5	0.249	49.7±6.1	50.7±6.9	51.5±7.0	50.3±6.9	50.4±6.7	0.103
BMI (kg/m^2^)	25.1±3.5	26.1±3.9	26.4±4.4	25.7±3.6	25.6±3.1	0.035	25.2±2.8	26.1±3.7	26.2±3.8	25.7±3.3	25.4±3.7	0.111
Waist circumference (cm)												
*men*	90.8±9.4	92.6±10.3	92.8±12.2	90.8±10.3	89.6±8.2	0.021	91.2±9.8	93.9±10.6	93.7±9.6	92.1±9.3	90.5±10.7	0.045
*women*	75.9±13.9	80.5±17.6	82.5±13.3	75.2±9.0	75.2±10.9	0.04	N.O.	89.0±10.7	84.4±10.4	86.6±12.3	82.0±7.4	0.21
Total cholesterol (mmol/l)	6.2±1.2	6.3±1.2	6.1±1.1	6.0±1.2	6.0±1.1	0.065	6.1±1.3	6.0±1.1	5.8±1.1	6.0±1.0	5.9±1.2	0.122
HDL cholesterol (mmol/l)	1.32±0.3	1.29±0.4	1.33±0.4	1.34±0.4	1.34±0.3	0.668	1.14±0.3	1.24±0.3	1.21±0.3	1.23±0.3	1.16±0.3	0.331
Triglycerides (mmol/l)	1.5(1.0, 2.0)	1.7(1.0, 2.5)	1.4(1.0, 2.1)	1.3(1.0, 1.9)	1.3(0.9, 1.9)	0.001	1.8(1.1, 3.3)	1.7(1.3, 2.6)	1.8(1.0, 2.4)	1.7(1.1, 2.4)	2.0(1.1, 2.7)	0.461
Systolic BP (mmHg)	122.9±18.8	123.3±18.4	122.0±17.9	122.8±16.9	124.0±17.5	0.841	120.8±18.4	126.0±17.9	125.7±18.6	124.0±16.6	124.4±16.3	0.415
Diastolic BP (mmHg)	77.6±11.7	76.6±11.5	76.1±10.8	77.2±10.6	77.1±11.0	0.641	79.7±11.4	81.0±10.7	79.9±10.5	79.8±10.2	79.4±10.0	0.765
Hypertension or treated n(%)	15(25.0)	43(24.4)	73(21.0)	85(22.6)	26(19.9)	0.825	9(28.1)	56(32.9)	157(32.6)	86(31.9)	16(30.2)	0.979
Hypertension treatment n(%)	5(8.3)	13(7.4)	29(8.4)	33(8.9)	7(5.3)	0.786	2(6.3)	23(13.5)	69(14.3)	37(13.7)	5(9.4)	0.646
Current smokers n(%)	60(100)	121(68.8)	129(37.2)	36(9.6)	0	0	32(100)	50(29.4)	37(7.7)	12(4.4)	0	0
Alcohol consumption (units/week)	34.9(0.5, 42.7)	13.2(0.3, 35.1)	10.3(0.9, 24.1)	8.5(3.1, 15.9)	8.0(4.5, 13.5)	0.001	0.1(0, 37.9)	0.1(0, 26.0)	0(0, 7.0)	4.0(0.3, 10.8)	7(3.3, 11.5)	0
Physical activity (MJ/week)	1.5(1.0, 4.0)	4.0(1.5, 5.0)	4.0(1.5, 6.6)	5.0(3.5, 10.2)	11.9(9.0, 15.8)	0	1.5(1.0, 3.5)	1.5(1.0, 4.0)	2.3(1.0, 4.0)	4.6(1.5, 9.0)	9.8(8.8, 11.6)	0
Fruit & vegetable (intake/week)	2.5(2.5, 3.5)	5(2.5, 6.5)	8(5, 11)	8(8, 11)	11(8, 11)	0	3.5(2, 5)	5(3.5, 5)	8(5.5, 11)	8(8, 11)	11(8, 11)	0
Manual social class n(%)	37(61.7)	118(67.1)	215(62.0)	202(53.7)	67(51.2)	0.008	25(78.1)	120(70.6)	360(74.8)	214(79.3)	41(77.4)	0.329
Unemployed n(%)	5(8.5)	26(14.8)	51(14.8)	46(12.3)	16(12.2)	0.609	8(25.0)	57(34.1)	133(27.7)	44(16.3)	5(9.6)	0
Diabetes n(%)	2(3.3)	9(5.1)	20(5.8)	22(5.9)	5(3.8)	0.841	5(15.6)	38(22.4)	111(23.1)	50(18.5)	10(18.9)	0.546

Values are shown as means (SD) for normally distributed continuous variables, medians (IQR) for non-normally distributed continuous variables, and numbers (percentages) for categorical variables.

* P value for difference using ANOVA, Kruskal-Wallis, or χ2, as appropriate.

The hazard ratios (HRs) (95% confidence intervals [CI]) of CVD and CHD significantly increased with decreasing number of health behaviours in each ethnic group (Table 2). Among Europeans, HRs for incident CVD by adherence to three, two, one, and zero compared to four health behaviours were 0.96 (0.61–1.51), 1.51 (0.97–2.34), 1.11 (0.67–1.84) and 2.12 (1.14–3.94) respectively in adjusted analyses, *p*-trend = 0.014. In South Asians, the equivalent HRs for incident CVD were of greater magnitude: 1.98 (1.02–3.81), 1.76 (0.92–3.36), 2.42 (1.23–4.74) and 2.73 (1.20–6.21) respectively, *p*-trend = 0.018. For CHD, among Europeans the HRs by adherence to three, two, one, and zero compared to four health behaviours were 1.33 (0.78–2.29), 1.96 (1.15–3.33), 1.36 (0.74–2.48) and 2.45 (1.18–5.10) respectively, *p*-trend = 0.025. The largest HRs were observed in South Asians for incident CHD of 2.88 (1.33–6.24), 2.28 (1.06–4.91), 3.36 (1.53–7.39) and 3.48 (1.38–8.81) respectively, *p*-trend = 0.022. These findings were unchanged in all sensitivity analyses and after testing for possible reverse causality (**S1A and S1B Table**).

**Table 2:** Hazard ratios (95% CI) of incident cardiovascular disease (CVD) and coronary heart disease (CHD) by number of health behaviours in Europeans and South Asians (without prevalent cardiovascular disease) using multivariable Cox regression models; the SABRE Study, UK.

CVD		Health behaviour score					
	Events/n	4	3	2	1	0	P value*
**Model 1**							
European	255/1090	1	0.97(0.62, 1.50)	1.50(0.98, 2.30)	1.28(0.79, 2.08)	2.12(1.16, 3.85)	0.003
South Asian	346/1006	1	1.88(1.03, 3.42)	1.59(0.88, 2.86)	2.16(1.17, 3.99)	2.40(1.11, 5.20)	0.046
**Model 2**							
European	243/1065	1	0.96(0.61, 1.51)	1.51(0.97, 2.34)	1.11(0.67, 1.84)	2.12(1.14, 3.94)	0.014
South Asian	328/970	1	1.98(1.02, 3.81)	1.76(0.92, 3.36)	2.42(1.23, 4.74)	2.73(1.20, 6.21)	0.018
							
**CHD**		**Health behaviour score**					
	Events/n	4	3	2	1	0	P value
**Model 1**							
European	207/1090	1	1.30(0.77, 2.20)	1.85(1.11, 3.09)	1.57(0.88, 2.78)	2.35(1.16, 4.73)	0.007
South Asian	313/1006	1	2.76(1.34, 5.69)	2.16(1.06, 4.42)	3.20(1.53, 6.67)	3.29(1.36, 7.95)	0.023
**Model 2**							
European	198/1065	1	1.33(0.78, 2.29)	1.96(1.15, 3.33)	1.36(0.74, 2.48)	2.45(1.18, 5.10)	0.025
South Asian	297/970	1	2.88(1.33, 6.24)	2.28(1.06, 4.91)	3.36(1.53, 7.39)	3.48(1.38, 8.81)	0.022

Model 1: adjusted for age and sex.

Model 2: additionally adjusted for BMI (kg/m^2^), diastolic blood pressure (mmHg), systolic blood pressure (mmHg), hypertension treatment (0 = no, 1 = yes), total cholesterol (mmHg), HDL cholesterol (mmHg), social class (1 = non-manual, 2 = manual), employment (0 = no, 1 = yes), and occupational physical activity (MJ/week, quartiles).

*P value for trend.

Comparisons of baseline characteristics of the total sample, those with follow-up data, and those without follow-up data showed no substantial variation (**S2 Table**). Results were of the same direction and greater magnitude when restricting analyses to only fatal CVD events (n = 264) in the total sample (n = 3,088) (**S3 Table**). We found no association (p = 0.985) between missing follow-up data and number of health behaviours using logistic regression (Model 2) in the total baseline sample (n = 3,358).

For individual health behaviours, smoking was positively associated with incident CVD and CHD in both ethnic groups (**Table 3**). A non-moderate alcohol intake significantly increased the HRs for incident CVD in Europeans and for incident CHD in South Asians.

**Table 3:** Hazard ratios (95%CI) of incident cardiovascular disease (CVD) and coronary heart disease (CHD) by individual health behaviors in Europeans and South Asians (without prevalent cardiovascular disease) using multivariable Cox regression models; the SABRE study, UK.

CVD		Model 3
events/total		243/1065
**European**		
	current smoker	1.41(1.06, 1.87)
	alcohol intake <1 and >14/21* units/week	1.27(0.98, 1.65)
	not physically active	1.11(0.84, 1.46)
	infrequent fruit or vegetable intake	0.92(0.69, 1.24)
events/total		328/970
**South Asian**		
	current smoker	1.63(1.21, 2.20)
	alcohol intake <1 and >14/21 units/week	1.26(0.99, 1.61)
	not physically active	0.93(0.71, 1.22)
	infrequent fruit or vegetable intake	1.03(0.81, 1.31)
**CHD**		Model 3
events/total		198/1065
**European**		
	current smoker	1.50(1.10, 2.05)
	alcohol intake <1 and >14/21 units/week	1.13(0.85, 1.51)
	not physically active	1.21(0.89, 1.64)
	infrequent fruit or vegetable intake	0.91(0.66, 1.26)
events/total		297/970
**South Asian**		
	current smoker	1.59(1.16, 2.18)
	alcohol intake <1 and >14/21 units/week	1.29(1.00, 1.67)
	not physically active	0.97(0.73, 1.29)
	infrequent fruit or vegetable intake	0.99(0.77, 1.28)

Model 3: adjusted for age, sex, BMI (kg/m^2^), diastolic blood pressure (mmHg), systolic blood pressure (mmHg), hypertension treatment (0 = no, 1 = yes), total cholesterol (mmHg), HDL cholesterol (mmHg), social class (1 = non-manual, 2 = manual), employment (0 = no, 1 = yes), OPA (MJ/week, quartiles) and mutually adjusted for the other health behaviours.

* women: 14 units/week, men 21 units/week.

The PAF for CVD incidence for adherence to the four combined health behaviours versus fewer was 28% among Europeans and 51% among South Asians. For CHD incidence, the PAF was 43% among Europeans and 63% among South Asians.

## Discussion

In this prospective study, lower adherence to a combination of healthy behaviours (non-smoker, moderate alcohol intake, physically active and frequent fruit and vegetable intake) was associated with increasing risk of incident CVD and CHD over 21 years of follow-up in both Europeans and South Asians in the UK. The impact on incident CVD outcomes by adherence to the health behaviours, calculated as the PAF, the proportion of events that could be prevented by eliminating the non-healthy behaviours in this population, was high in both Europeans (between 28–43%) and South Asians (51–63%). These findings for Europeans are consistent with past evidence [14, 15, 19], while the findings among South Asians are novel.

### Findings in context of other evidence

Compared with the UK EPIC-Norfolk study, we used comparable measures for smoking, but used a sex-specific alcohol definition, a stricter definition of being physically active, and a more limited self-reported diet measure, yet our estimates for incident CVD and CHD for Europeans are consistent with the EPIC-Norfolk findings for incident stroke [18]. Similarly, our findings for Europeans in Southall are consistent with findings in three American studies of health professionals [14–16].

The population impact we observed of a PAF of 43% for CHD (and 28% for CVD) in Europeans is lower than previously reported [14, 15]. However, the US-based studies estimated a population attributable risk (PAR) of incident CHD, comparing those with adherence to five health behaviours with the rest of the population, translating to a PAR of 62% in male health professionals [15] and a PAR of 82% in The Nurses’ Health Study [14], while our method enabled us to account for each level of adherence to the health behaviour score.

The populations in other published studies are dominated by health professionals [14–16] whereas our findings show that these associations are also apparent in manual occupations. The CVD incidence rates we observed in both Europeans and South Asians from West London was higher than rates reported for US Health Professional men [15] and adults from the UK Norfolk-based study [37]. The greater magnitude of the disease burden in the current population implies the potential for risk reductions especially in South Asians, where 95% of the population could improve their health behaviours and if everyone adhered to the four health behaviours, 51–63% of CVD events could be prevented.

With regard to single health behaviours, we found smoking to be a strong risk factor both for incident CVD and CHD, consistent with previous evidence in Europeans [14–16, 19] and South Asians [22, 38]. Non-moderate alcohol intake was associated with increased risks of CVD in Europeans and CHD in South Asians. However, the distribution of adherence to this health behaviour varied substantially by ethnicity; non-moderate alcohol intake in Europeans was mostly due to high intakes (n = 273, 25% of total), whereas in South Asians, non-adherence was mostly due to abstaining (n = 420, 42% of total) as previously reported [32]. We found a tendency (not significant) towards increasing CVD and CHD incidence with being inactive among Europeans, but among South Asians there was a null association. Our finding of South Asians being less physically active than Europeans is consistent with previous reports [39, 40]. A comprehensive dietary score [14, 15] and plasma vitamin C levels [18, 19], as indicators of a healthy diet behaviour, have previously reported to be associated with decreased risk of CVD related outcomes, while the fruit and vegetable intake health behaviour in the current analysis was not.

### Limitations and strengths

The loss to follow-up (29%) was a potential source of bias in this study, but three separate sets of analyses confirmed this was not substantial. We compared baseline characteristics among participants with and without follow-up data, we restricted analyses to fatal CVD only (since everyone was traced for mortality), and we found no association between having missing follow-up data and number of health behaviours at baseline. With a single time-point assessment of self-reported health behaviours we cannot account for potential changes in health behaviours during the study period. By dichotomising each of the four health behaviours and weighting the healthier behaviours equally, we did not allow for differential importance or potential dose-response relationships of the individual health behaviours. Physical activity was measured using a questionnaire, which was limited in differentiation of intensity, and we were unable to include occupational physical activity in the activity health behaviour due to limited information, though we did account for occupational physical activity in our multivariable model. The cut-off for being classified as physically active corresponds to about 30 minutes of vigorous activity per day which is higher than the recommendation of 30 minutes of moderate to vigorous activity a day [41]. Nevertheless previous studies have used different definitions of being physically active [19, 33, 42, 43], and 37% of Europeans and 23% of South Asians met the cut-off of 3kcal/kg/day in our study, which is consistent with an earlier study comparing ethnic groups [33]. Our higher cut-off of qualifying as being physically active was expected to show a strong relationship between physical activity and CVD outcomes. The dietary questionnaire we used was limited by being simple, not validated and did not enable estimation of total energy intake. However, we were able to include fruit and vegetable intake frequency, widely used as a marker of healthy diet [10, 12, 13]. Our non-significant findings for the physical activity and the fruit and vegetable intake health behaviour, are possibly explained by measurement error, recall bias and social desirability bias. Misclassification of participants to the healthy behaviours would result in an underestimation of the true effect of the health behaviour and our estimates would therefore be conservative. Religious practice could be an important factor in determining some health behaviours (such as non-consumption of alcohol among Muslim participants or not smoking among Sikh participants); adjustment for religion did not materially influence our findings (results not shown). Our findings are based on first-generation immigrants, therefore they may not apply to all South Asians. However, emerging studies on UK born South Asians have indicated the importance of health behaviours in second generation South Asians [44, 45]. We note that our definition of CVD included CHD and stroke events in line with our previous work [26] and we did not include peripheral vascular disease events as these data were very limited on GP record review. Finally we cannot quantify the completeness of our case ascertainment with ONS linkage for mortality, but we would not expect completeness to differ by ethnicity and also any under-reporting would tend to bias the results towards the null, therefore our estimates might be conservative. Our use of the direct method of primary care data review for events by two senior physicians blinded to participant ethnicity and identity has, however, enhanced case ascertainment.

The large population-based cohort of both Europeans and South Asians and long follow-up period in this study provides unique prospective ethnicity-specific data on incident CVD outcomes. The estimation and measurement of a wide variety of variables at baseline allowed for adjustment for many possible covariates and our findings were robust to a range of adjustments and sensitivity analyses. The PAFs reported in this study provide novel information that can be used in public health messages for CVD prevention. Among Europeans, previously only population impacts for incident CHD, or for mortality but not incident CVD, have been reported. In South Asians, the impact of a composite health behaviour score on CVD or CHD incidence was previously unknown.

### Implications and key messages

To our knowledge this study is the first to investigate the role of combined health behaviours on risk of CVD and CHD and their population impact in South Asian immigrants in the UK compared to a European population. Though South Asians in the UK have an excess risk of CVD and CHD compared with Europeans, the risk reduction by adherence to a healthy lifestyle is also of great importance in South Asians, as was shown previously in European populations but was unknown for South Asians. Increasing physical activity among South Asians has the greatest potential for prevention strategies underlined by the striking disparity in this health behaviour. From a public health perspective, to target and inform this specifically high-risk group about health behaviours should be prioritised in order to reduce the burden of CVD events.

## Conclusions

Lack of adherence to four combined health behaviours was associated with 2 to 3-fold increased risk of incident CVD and CHD in both South Asians and Europeans living in the same area of London, UK. In this study population between 51 to 63% of CVD and CHD events in South Asians and 28 to 43% in Europeans could have been prevented if all participants had adhered to the combined health behaviours. Although CVD risk is increased among South Asian UK immigrants compared to the general UK population, adherence to a healthy lifestyle has a substantial impact in this group as well as among Europeans in the UK.

### Data

All data necessary to replicate these analyses and to draw conclusions are contained in the anonymised supporting information files.

## Supporting Information

S1 Data (XLSX)

S1 Table S1A: Sensitivity analyses; hazard ratios (95%CI) of incident cardiovascular disease by number of health behaviours in Europeans and South Asians (without prevalent cardiovascular disease) using multivariable Cox regression; the SABRE Study, UK. S1B: Sensitivity analyses; hazard ratios (95%CI) of incident coronary heart disease by number of health behaviours in Europeans and South Asians (without prevalent cardiovascular disease) using multivariable Cox regression; the SABRE Study, UK
(DOCX)

S2 Table Baseline characteristics by follow-up status for Europeans and South Asians; the SABRE Study, UK. (DOCX)

S3 Table Hazard ratios (95% CI) of fatal cardiovascular disease by number of health behaviours in multivariable adjusted models in the total sample of Europeans and South Asians (without prevalent cardiovascular disease); the SABRE Study, UK. (DOCX)
