# Supplementary material for: The Impact of Health Behaviours on Incident Cardiovascular Disease in Europeans and South Asians – A Prospective Analysis in the UK SABRE Study
Source: PLoS One. 2015 Mar 2;10(3):e0117364. doi: 10.1371/journal.pone.0117364 (PMC4346401; doi:10.1371/journal.pone.0117364)
Supplement: S1 Table — S1B: Sensitivity analyses; hazard ratios (95%CI) of incident coronary heart disease by number of health behaviours in Europeans and South Asians (without prevalent cardiovascular disease) using multivariable Cox regression; the SABRE Study, UK (DOCX) [file pone.0117364.s002.docx]

| **Table S1A.** Sensitivity analyses; hazard ratios (95%CI) of incident cardiovascular disease by number of health behaviours in Europeans and South Asians (without prevalent cardiovascular disease) using multivariable Cox regression; the SABRE Study, UK | | | | | | | | |
| --- | --- | --- | --- | --- | --- | --- | --- | --- |
|  |  | Health behaviour score | | | | | |  |
|  |  | 4 | 3 | | 2 | 1 | 0 | P value* |
| **Model 2** |  |  |  | |  |  |  |  |
| European | 243/1065 | 1 | 0.96(0.61, 1.51) | | 1.51(0.97, 2.34) | 1.11(0.67, 1.84) | 2.12(1.14, 3.94) | 0.014 |
| South Asian | 328/970 | 1 | 1.98(1.02, 3.81) | | 1.76(0.92, 3.36) | 2.42(1.23, 4.74) | 2.73(1.20, 6.21) | 0.018 |
| **Model 2 +** |  |  |  | |  |  |  |  |
| **Years of education** | |  |  | |  |  |  |  |
| European | 240/1059 | 1 | 0.96(0.61, 1.51) | | 1.49(0.96, 2.34) | 1.11(0.66, 1.85) | 1.98(1.05, 3.75) | 0.021 |
| South Asian | 300/900 | 1 | 1.91(0.99, 3.70) | | 1.77(0.93, 3.39) | 2.31(1.17, 4.55) | 2.69(1.18, 6.13) | 0.021 |
| **Education without social class** | | |  | |  |  |  |  |
| European | 240/1059 | 1 | 0.95(0.60, 1.51) | | 1.48(0.95, 2.32) | 1.10(0.66, 1.83) | 1.97(1.04, 3.71) | 0.024 |
| South Asian | 300/900 | 1 | 1.91(0.98, 3.69) | | 1.77(0.93, 3.39) | 2.32(1.18, 4.56) | 2.69(1.18, 6.13) | 0.020 |
| **Diabetes** |  |  |  | |  |  |  |  |
| European | 243/1065 | 1 | 0.95(0.60, 1.49) | | 1.49(0.96, 2.31) | 1.10(0.66, 1.83) | 2.09(1.12, 3.88) | 0.015 |
| South Asian | 328/970 | 1 | 2.15(1.11, 4.16) | | 1.84(0.96, 3.50) | 2.60(1.32, 5.10) | 3.20(1.41, 7.29) | 0.011 |
| **Triglycerides** |  |  |  | |  |  |  |  |
| European | 243/1065 | 1 | 0.97(0.62, 1.53) | | 1.52(0.98, 2.35) | 1.09(0.66, 1.81) | 2.13(1.14, 3.97) | 0.017 |
| South Asian | 328/970 | 1 | 2.05(1.05, 3.99) | | 1.82(0.94, 3.50) | 2.49(1.26, 4.93) | 2.82(1.23, 6.47) | 0.017 |
| **Waist circumference** | |  |  | |  |  |  |  |
| European | 243/1065 | 1 | 0.95(0.60, 1.49) | | 1.47(0.94, 2.28) | 1.07(0.64, 1.79) | 2.04(1.09, 3.81) | 0.024 |
| South Asian | 328/970 | 1 | 1.98(1.03, 3.83) | | 1.77(0.93, 3.37) | 2.44(1.24, 4.79) | 2.74(1.21, 6.23) | 0.018 |
| **Waist:hip** |  |  |  | |  |  |  |  |
| European | 243/1065 | 1 | 0.95(0.61, 1.50) | | 1.43(0.92, 2.22) | 1.04(0.62, 1.73) | 1.93(1.03, 3.62) | 0.049 |
| South Asian | 328/970 | 1 | 1.93(1.00, 3.72) | | 1.71 (0.89, 3.25) | 2.30(1.17, 4.52) | 2.69(1.19, 6.12) | 0.026 |
| **Excluding participants with events in the first 2 years** | | | | | |  |  |  |
| European | 229/1051 | 1 | 0.96(0.60, 1.52) | 1.47(0.94, 2.31) | | 1.07(0.63, 1.80) | 2.13(1.13, 4.03) | 0.023 |
| South Asian | 292/934 | 1 | 1.93(0.96, 3.86) | 1.68(0.85, 3.32) | | 2.41(1.18, 4.90) | 3.13(1.34, 7.30) | 0.011 |
| **Excluding participants with events in the first 5 years** | | | | | |  |  |  |
| European | 195/1017 | 1 | 0.95(0.58, 1.55) | 1.46(0.90, 2.35) | | 0.91(0.51, 1.63) | 1.95(0.97, 3.91) | 0.106 |
| South Asian | 252/894 | 1 | 1.63(0.81, 3.29) | 1.49(0.75, 2.96) | | 1.96(0.95, 4.04) | 3.03(1.28, 7.19) | 0.023 |
| Model 2: adjusted for age (y, continuous), sex, BMI (kg/m^2^, continuous), diastolic blood pressure (mmHg, continuous), systolic blood pressure (mmHg, continuous), hypertension treatment (0=no, 1=yes), total cholesterol (mmHg, continuous), HDL cholesterol (mmHg, continuous), social class (1=non-manual, 2=manual), employment (0=no, 1=yes), and occupational physical activity (MJ/week, quartiles) *P value for trend | | | | | | | | |

| **Table S1B.** Sensitivity analyses; hazard ratios (95%CI) of incident coronary heart disease by number of health behaviours in Europeans and South Asians (without prevalent cardiovascular disease) using multivariable Cox regression; the SABRE Study, UK | | | | | | | | | | |
| --- | --- | --- | --- | --- | --- | --- | --- | --- | --- | --- |
|  |  | | Health behaviour score | | | | | | |  |
|  |  | | 4 | | 3 | | 2 | 1 | 0 | P value* |
| **Model 2** |  | |  | |  | |  |  |  |  |
| European | 198/1065 | | 1 | | 1.33(0.78, 2.29) | | 1.96(1.15, 3.33) | 1.36(0.74, 2.48) | 2.45(1.18, 5.10) | 0.025 |
| South Asian | 297/970 | | 1 | | 2.88(1.33, 6.24) | | 2.28(1.06, 4.91) | 3.36(1.53, 7.39) | 3.48(1.38, 8.81) | 0.022 |
| **Model 2 +** |  | |  | |  | |  |  |  |  |
| **Years of education** | | |  | |  | |  |  |  |  |
| European | 195/1059 | | 1 | | 1.35(0.78, 2.35) | | 1.99(1.15, 3.43) | 1.38(0.75, 2.55) | 2.29(1.07, 4.91) | 0.037 |
| South Asian | 272/900 | | 1 | | 2.79(1.28, 6.07) | | 2.31(1.07, 4.98) | 3.22(1.46, 7.11) | 3.46(1.36, 8.75) | 0.025 |
| **Education without social class** | | | | |  | |  |  |  |  |
| European | 195/1059 | | 1 | | 1.34(0.77, 2.33) | | 1.96(1.14, 3.38) | 1.35(0.73, 2.50) | 2.24(1.05, 4.79) | 0.045 |
| South Asian | 272/900 | | 1 | | 2.78(1.28, 6.04) | | 2.32(1.08, 5.00) | 3.24(1.47, 7.17) | 3.46(1.36, 8.76) | 0.022 |
| **Diabetes** |  | |  | |  | |  |  |  |  |
| European | 198/1065 | | 1 | | 1.31(0.76, 2.25) | | 1.93(1.14, 3.29) | 1.34(0.73, 2.46) | 2.40(1.15, 5.01) | 0.028 |
| South Asian | 297/970 | | 1 | | 3.09(1.42, 6.70) | | 2.35(1.09, 5.05) | 3.53(1.61, 7.77) | 3.88(1.53, 9.82) | 0.019 |
| **Triglycerides** |  | |  | |  | |  |  |  |  |
| European | 198/1065 | | 1 | | 1.36(0.79, 2.34) | | 1.99(1.17, 3.38) | 1.34(0.74, 2.46) | 2.46(1.18, 5.14) | 0.029 |
| South Asian | 297/970 | | 1 | | 3.00(1.37, 6.60) | | 2.38(1.09, 5.16) | 3.49(1.57, 7.75) | 3.63(1.42, 9.26) | 0.022 |
| **Waist circumference** | | |  | |  | |  |  |  |  |
| European | 198/1065 | | 1 | | 1.31(0.76, 2.25) | | 1.90(1.11, 3.24) | 1.30(0.71, 2.39) | 2.32(1.11, 4.87) | 0.046 |
| South Asian | 297/970 | | 1 | | 2.90(1.34, 6.29) | | 2.30(1.07, 4.95) | 3.41(1.55, 7.51) | 3.50(1.38, 8.86) | 0.021 |
| **Waist:hip** |  | |  | |  | |  |  |  |  |
| European | 198/1065 | | 1 | | 1.33(0.77, 2.28) | | 1.86(1.09, 3.17) | 1.27(0.70, 2.33) | 2.21(1.05, 4.63) | 0.075 |
| South Asian | 297/970 | | 1 | | 2.83(1.30, 6.14) | | 2.24(1.04, 4.81) | 3.24(1.47, 7.14) | 3.46(1.37, 8.75) | 0.029 |
| **Excluding participants with events in the first 2 years** | | | | | | | |  |  |  |
| European | 185/1052 | 1 | | 1.35(0.78, 2.36) | | 1.97(1.14, 3.40) | | 1.32(0.70, 2.46) | 2.47(1.15, 5.28) | 0.039 |
| South Asian | 267/940 | 1 | | 2.97(1.29, 6.85) | | 2.30(1.01, 5.26) | | 3.60(1.54, 8.39) | 4.17(1.57, 11.07) | 0.010 |
| **Excluding participants with events in the first 5 years** | | | | | | | |  |  |  |
| European | 156/1023 | 1 | | 1.44(0.78, 2.66) | | 2.18(1.19, 3.98) | | 1.23(0.60, 2.49) | 2.28(0.96, 5.39) | 0.113 |
| South Asian | 233/906 | 1 | | 2.66(1.15, 6.16) | | 2.09(0.91, 4.79) | | 3.13(1.33, 7.36) | 4.04(1.50, 10.89) | 0.018 |
| Model 2: adjusted for age (y, continuous), sex, BMI (kg/m^2,^, continuous), diastolic blood pressure (mmHg, continuous), systolic blood pressure (mmHg, continuous), hypertension treatment (0=no, 1=yes), total cholesterol (mmHg, continuous), HDL cholesterol (mmHg, continuous), social class (1=non-manual, 2=manual), employment (0=no, 1=yes), and occupational physical activity (MJ/week, quartiles) *P value for trend | | | | | | | | | | |
