# Supplementary material for: The Impact of Health Behaviours on Incident Cardiovascular Disease in Europeans and South Asians – A Prospective Analysis in the UK SABRE Study
Source: PLoS One. 2015 Mar 2;10(3):e0117364. doi: 10.1371/journal.pone.0117364 (PMC4346401; doi:10.1371/journal.pone.0117364)
Supplement: S2 Table — (DOCX) [file pone.0117364.s003.docx]

| **Table S2.** Baseline characteristics by follow-up status for Europeans and South Asians; the SABRE Study, UK | | | | | | | |
| --- | --- | --- | --- | --- | --- | --- | --- |
|  |  | Total sample | | Followed-up | | Lost to follow-up | |
| Variable | | European | South Asian | European | South Asian | European | South Asian |
| n (%men) | | 1761(86) | 1710(83) | 1235(89) | 1148(84) | 526(80) | 562(80) |
| Age (years) | | 53.0±7.2 | 51.1±7.0 | 53.4±7.3 | 51.2±7.0 | 52.3±6.9 | 50.7±6.9 |
| BMI (kg/m2) | | 26.1±3.9 | 26.1±3.6 | 26.0±3.9 | 26.0±3.6 | 26.2±4.0 | 26.3±3.6 |
| Waist circumference (cm) | |  |  |  |  |  |  |
|  | men | 91.8±10.9 | 93.3±9.6 | 91.8±10.9 | 93.1±9.6 | 91.9±11.1 | 93.6±9.7 |
|  | women | 78.8±12.4 | 85.8±11.0 | 79.0±12.7 | 85.8±11.1 | 78.6±12.2 | 85.8±10.9 |
| Total cholesterol (mmol/l) | | 6.1±1.2 | 5.9±1.1 | 6.2±1.2 | 6.0±1.1 | 6.1±1.1 | 5.9±1.0 |
| HDL cholesterol (mmol/l) | | 1.3±0.4 | 1.2±0.3 | 1.3±0.4 | 1.2±0.3 | 1.3±0.4 | 1.2±0.4 |
| Triglycerides (mmol/l) | | 1.4(1, 2.1) | 1.7(1.2, 2.5) | 1.4(1.0, 2.1) | 1.7(1.2, 2.5) | 1.4(1.0, 2.0) | 1.7(1.1, 2.4) |
| Systolic BP (mmHg) | | 122.9±17.2 | 125.3±18.0 | 122.8±17.7 | 125.6±18.4 | 123.0±16.0 | 124.8±17.3 |
| Diastolic BP (mmHg) | | 76.9±10.7 | 80.2±10.6 | 76.7±10.8 | 80.2±10.5 | 77.5±10.3 | 80.1±10.8 |
| Hypertension or treated n(%) | | 408(23) | 550(32) | 298(24) | 389(34) | 110(21) | 161(29) |
| Current smokers n(%) | | 553(31) | 232(14) | 394(32) | 162(14) | 159(30) | 70(13) |
| Alcohol consumption (units/week) | | 8.3(1.6, 20.0) | 0.8(0, 12) | 9.1(1.7, 20.8) | 0.9(0, 12) | 7.2(1.6, 18.1) | 0.3(0, 11.5) |
| Physical activity (MJ/week) | | 4.0(1.5, 9.8) | 3.5(1, 5.3) | 4.0(1.7, 9.6) | 3.5(1, 5.3) | 4.0(1.5, 9.9) | 3.5(1, 6.0) |
| Fruit & vegetable (intake/week) | | 7.2±3.3 | 7.7±3.1 | 7.3±3.2 | 7.6±3.1 | 6.9±3.4 | 7.7±3.2 |
| Health behaviours n(%) | |  |  |  |  |  |  |
|  | 0 | 99(6) | 52(3) | 68(6) | 37(3) | 31(6) | 15(3) |
|  | 1 | 297(17) | 307(18) | 203(16) | 205(18) | 94(18) | 102(18) |
|  | 2 | 576(33) | 807(47) | 406(33) | 537(47) | 170(32) | 270(48) |
|  | 3 | 581(33) | 448(26) | 417(34) | 310(27) | 164(31) | 138(25) |
|  | 4 | 207(12) | 91(5) | 140(11) | 58(5) | 67(13) | 33(6) |
| Manual social class n(%) | | 1083(62) | 1311(77) | 745(60) | 875(76) | 338(64) | 436(78) |
| Occupational physical activity (MJ/week) | | 5(0, 7) | 5(0,8) | 5(2, 7) | 5(0, 8) | 5(3, 7) | 6(0, 8) |
| Unemployed n(%) | | 271(15) | 432(26) | 190(15) | 297(26) | 81(15) | 135(24) |
| Diabetes n(%) | | 97(6) | 355(21) | 78(6) | 267(23) | 19(4) | 88(16) |
| Data presented as mean (SD) or median (IQR) for continuous variables and numbers (percentages) for categorical variables | | | | | | | |
