# Supplementary material for: The Impact of Health Behaviours on Incident Cardiovascular Disease in Europeans and South Asians – A Prospective Analysis in the UK SABRE Study
Source: PLoS One. 2015 Mar 2;10(3):e0117364. doi: 10.1371/journal.pone.0117364 (PMC4346401; doi:10.1371/journal.pone.0117364)
Supplement: S3 Table — (DOCX) [file pone.0117364.s004.docx]

| **Table S3.** Hazard ratios (95% CI) of fatal cardiovascular disease by number of health behaviours in multivariable adjusted models in the total sample of Europeans and South Asians (without prevalent cardiovascular disease); the SABRE Study, UK | | | | | | | |
| --- | --- | --- | --- | --- | --- | --- | --- |
| **CVD** |  | **Health behaviour score** | | | | |  |
|  | Events/n | 4 | 3 | 2 | 1 | 0 | P value |
| **Model 1** |  |  |  |  |  |  |  |
| European | 117/1580 | 1 | 1.06(0.49, 2.27) | 2.29(1.13, 4.67) | 2.74(1.29, 5.84) | 3.17(1.22, 8.26) | <0.001 |
| South Asian | 147/1508 | 1 | 4.86(1.17, 20.12) | 5.15(1.26, 21.01) | 5.81(1.38, 24.46) | 4.91(0.90, 26.83) | 0.059 |
| **Model 2** |  |  |  |  |  |  |  |
| European | 111/1546 | 1 | 1.16(0.52, 2.61) | 2.49(1.17, 5.29) | 2.98(1.34, 6.64) | 3.19(1.13, 8.94) | <0.001 |
| South Asian | 139/1455 | 1 | 8.62(1.18, 63.02) | 9.01(1.25, 65.08) | 10.44(1.41, 77.32) | 9.42(1.05, 84.82) | 0.047 |
| Model 1: adjusted for age (y, continuous) and sex; Model 2: additionally adjusted for BMI (kg/m^2^, continuous), diastolic blood pressure (mmHg, continuous), systolic blood pressure (mmHg, continuous), hypertension treatment (0=no, 1=yes), total cholesterol (mmHg, continuous), HDL cholesterol (mmHg, continuous), social class (1=non-manual, 2=manual), employment (0=no, 1=yes), and occupational physical activity (MJ/week, quartiles) * P value for trend | | | | | | | |
